# Risk Factors Associated With Boys’ and Girls’ Developmental Trajectories of Physical Aggression From Early Childhood Through Early Adolescence

**DOI:** 10.1001/jamanetworkopen.2018.6364

**Published:** 2018-12-28

**Authors:** Ali Teymoori, Sylvana M. Côté, Bobby L. Jones, Daniel S. Nagin, Michel Boivin, Frank Vitaro, Massimiliano Orri, Richard E. Tremblay

**Affiliations:** 1 Institute of Medical Psychology and Medical Sociology, University Medical Center Göttingen (UMG), Göttingen, Germany; 2 School of Public Health, University of Montreal, Montreal, Quebec, Canada; 3 Bordeaux Population Health Research Centre, Institut National de la Santé et de la Recherche Médicale (INSERM) U1219, University of Bordeaux, Bordeaux, France; 4 Department of Psychiatry, University of Pittsburgh, Pittsburgh, Pennsylvania; 5 Heinz College, Carnegie Mellon University, Pittsburgh, Pennsylvania; 6 School of Psychology, University Laval, Quebec, Quebec, Canada; 7 School of Psychoeducation, University of Montreal, Montreal, Quebec, Canada; 8 Department of Pediatrics, University of Montreal, Montreal, Quebec, Canada; 9 Department of Psychology, University of Montreal, Montreal, Quebec, Canada; 10 School of Public Health, University College Dublin, Dublin, Ireland

## Abstract

**Importance:**

This study used multitrajectory modeling to identify distinct trajectories of physical aggression from ages 1.5 to 13 years for boys and girls.

**Objectives:**

To trace the development of boys’ and girls’ physical aggression problems from infancy to adolescence using mother ratings, teacher ratings, and self-ratings and to identify early family predictors of children on the high physical aggression trajectories.

**Design, Setting, and Participants:**

This cohort study used data from the Quebec Longitudinal Study of Child Development (QLSCD), a study of a representative, population-based sample of 2223 infants born in 1997 and 1998 in the Canadian province of Quebec. The dates of analysis were January 2017 to January 2018.

**Main Outcomes and Measures:**

Trained research assistants conducted 7 interviews (at child ages 1.5, 2.5, 3.5, 4.5, 5, 6, and 8 years) with the person most knowledgeable about the child (mothers in 99.6% [2214 of 2223] of cases). Teachers assessed the child’s behavior at ages 6, 7, 8, 10, 12, and 13 years. Self-reports of behavior problems were obtained from the child at ages 10, 12, and 13 years.

**Results:**

The sample included 2223 participants, 51.2% of whom were boys and 91.2% of whom were of white race/ethnicity. The mean response rate for mother ratings of physical aggression during the first 8 years of life was 80.9% (range, 65.1%-91.7%). For teacher ratings of physical aggression from ages 6 to 13 years, the mean response rate was 45.7% (range, 35.4%-56.9%), while the mean response rate of physical aggression assessment from self-ratings between ages 10 and 13 years was 57.9% (range, 55.2%-60.5%). Attrition was higher among families with low socioeconomic status and single-parent families, as well as among young mothers and mothers who were not fluent in French or English. A statistical analysis to examine the consequences of attrition was included. For boys and girls, the frequency of physical aggressions increased from age 1.5 years (2039 [91.7%]) to age 3.5 years (1941 [87.3%]) and then substantially decreased until age 13 years (1228 [55.2%]). Three distinct developmental trajectories of physical aggression were observed for girls and 5 for boys. Most family characteristics measured at 5 months after the child’s birth were associated with a high physical aggression trajectory for boys and girls.

**Conclusions and Relevance:**

Family characteristics at 5 months after the child’s birth could be used to target preschool interventions aimed at preventing the development of boys’ and girls’ chronic physical aggression problems.

## Key Points

**Question:**

What are the physical aggression trajectories from ages 1.5 to 13 years and their risk factors when simultaneously taking into account mother ratings, teacher ratings, and self-ratings?

**Findings:**

In this cohort study using multitrajectory modeling for population samples of 2223 boys and girls, frequency of physical aggressions increased from ages 1.5 to 3.5 years and then substantially decreased. Three distinct developmental trajectories were observed for girls and 5 for boys, while several risk factors for the high physical aggression trajectories were identified.

**Meaning:**

Developmental trajectories of physical aggression differ for boys and girls, and distinct early risk factors could be targeted for preventive interventions.

## Teaser

This cohort study traces the development of boys’ and girls’ physical aggression problems from infancy to adolescence using mother ratings, teacher ratings, and self-ratings and identifies early family predictors of children on the high physical aggression trajectories among children.

## Introduction

The systematic study of the development of physical aggression from early childhood onward is recent. Approximately 20 years ago, reports by the Surgeon General of the United States^1^ and the World Health Organization^2^ concluded that most violent adolescents initiated their aggressive behavior during adolescence. However, large longitudinal studies^3,4,5,6,7,8,9^ from early childhood to midadolescence have shown that children start to physically aggress during the first 2 years after birth and reach a peak in frequency between ages 2 and 4 years. In most cases, this high frequency of physical aggressions declines before children enter elementary school according to parent reports and continues to decline thereafter according to teacher and self-reports.^3,4,5,10^ However, a small proportion of children maintain an atypically high frequency of physical aggressions during middle childhood and adolescence.^11^ This high physical aggression trajectory is associated with a range of negative outcomes in adolescence and adulthood, such as increased risk of violent crime,^5^ school maladjustment and school failure,^12,13^ alcohol and drug abuse,^14,15^ and social maladjustment.^14,15^

To prevent children from following a pathway of high physical aggression, we need to identify early family and educational risk factors contributing to high physical aggression.^16,17,18^ A major impediment in tracing the developmental trajectories of physical aggression from infancy to adolescence has been the lack of a uniform source of information. Observational studies are sometimes used in early childhood investigations,^19,20^ but they are too labor intensive for large,e population-based longitudinal studies. Self-reports cannot be used before the end of elementary school.^21^ The mother is the main source of information during early childhood and early elementary school,^4,10^ teachers are the main sources of information during the elementary school years,^3,5,22,23,24^ and children’s self-reports are the preferred source of information from age 10 years onward.^21^

Therefore, to trace the development of physical aggression from early childhood to adolescence so as to understand changes across distinct developmental phases (early childhood, childhood, and adolescence), we must rely on different raters (usually mothers, teachers, and self-reports). To our knowledge, no longitudinal study has integrated these different sources of information, mainly because of the limits imposed by the available statistical tools to trace the developmental trajectories.

Longitudinal studies^3,9,25,26,27,28^ mapping the development of physical aggression have shown important sex differences, with boys using physical aggression more often than girls. Boys are also more likely than girls to comprise a chronic physical aggression group.^3,7^ However, most studies quantifying sex differences in physical aggression have relied on a single source of information.

Early risk factors for chronic physical aggression have been identified with longitudinal studies of physical aggression using a developmental trajectory approach based on a sole source of information. These risk factors include the presence of siblings, young maternal age at first birth, parents’ low education, low socioeconomic status, and parents’ high level of antisocial behavior during their own adolescence.^4,5,6^ Integrating the perspectives of mothers, teachers, and children on the development of physical aggression from early childhood to early adolescence might lead to different forms of trajectories and different sex differences over time, as well as differences in the early childhood predictors of the chronic physical aggression trajectories.

The present study used group-based multitrajectory modeling^29^ to identify the following: (1) the developmental trajectories of physical aggression from infancy to early adolescence based on mother ratings, teacher ratings, and self-ratings; (2) sex differences in the developmental trajectories; and (3) early risk factors for high physical aggression trajectories. Given that different raters observe physical aggression at different ages, in different contexts, and from different perspectives, the integration of multiple assessments in a multitrajectory analysis should provide a more comprehensive picture of the development of physical aggression.^30^

## Methods

This cohort study was approved by the Quebec Statistical Institute (Montreal, Canada) Ethics Committee. After describing the aim of the study, written informed consent was obtained from a parent of the child and/or from the child at each wave of data collection.

### Participants

We used data from the Quebec Longitudinal Study of Child Development (QLSCD), a cohort study of a representative, population-based sample of 2223 infants born in 1997 and 1998 in the Canadian province of Quebec. The dates of analysis were January 2017 to January 2018. Trained research assistants at the Quebec Statistical Institute conducted 7 interviews (at child ages 1.5, 2.5, 3.5, 4.5, 5, 6, and 8 years) with the person most knowledgeable about the child (mothers in 99.6% [2214 of 2223] of cases). Teachers assessed the child’s behavior at ages 6, 7, 8, 10, 12, and 13 years. Finally, self-reports of behavior problems were obtained from the child at ages 10, 12, and 13 years. The mean response rate for mother ratings of physical aggression during the first 8 years of life was 80.9% (range, 65.1%-91.7%). For teacher ratings of physical aggression from ages 6 to 13 years, the mean response rate was 45.7% (range, 35.4%-56.9%), while the mean response rate of physical aggression assessment from self-ratings between ages 10 and 13 years was 57.9% (range, 55.2%-60.5%).

Attrition was higher among families with low socioeconomic status and single-parent families, as well as among young mothers and mothers who were not fluent in French or English.^31^ Attrition analyses were conducted: we compared the demographic characteristics of children missing the last 2 self-report assessments (ages 12 and 13 years) with those who participated in the last 2 assessments. Using this method, 815 (36.7%) were categorized as missing and 1408 (63.3%) as participating. The missing group had significantly lower socioeconomic status at 5 months after the child’s birth (mean [SD], −0.11 [0.04]) compared with the participating group (mean [SD], 0.06 [0.03]) (*t* = 3.63, *P* < .001).

In multitrajectory modeling, there is no constraint on the number of missing observations. Therefore, we included a condition requiring participants to have at least 10 observations across the 16 observations to be included in the estimation sample. We also performed a second data analysis without the use of the constraint on the number of observations per participant to examine if the results differed notably in terms of the size and shape of the trajectories.

### Physical Aggression

The same items defining physical aggression were used from ages 1.5 to 13 years: (1) “gets into fights,” (2) “physically attacks others,” and (3) “hits, bites, kicks other children.” The respondents rated the items on a 3-point Likert-type scale as follows: the child never (1 point), sometimes (2 point), or often (3 points) exhibited the specific form of physical aggression. The α levels for each of the raters at each time point on these 3 items were within the acceptable range of 0.62 to 0.78.

### Demographic Information

During the first interview with the person most knowledgeable about the child, we obtained the following demographic information: sex, parents’ education (ranging from 1 [primary education] to 7 [graduate studies]), household income (9 groups ranging from<$1000 to>$80 000 per year), mother’s age at first childbirth, family structure (intact or not), parents’ depression (a short version of the Center for Epidemiologic Studies Depression Scale, including 12 items), socioeconomic status (an aggregate of annual gross income, parental education, and parents’ occupation),^32^ number of siblings at age 5 months and at age 17 months, and parents’ antisocial behavior during their own adolescence. All questionnaires are available online (http://www.jesuisjeserai.stat.gouv.qc.ca).

### Statistical Analysis

A statistical analysis to examine the consequences of attrition was included. We used descriptive statistics, *t* tests, logistic regression, and group-based trajectory modeling to analyze the data. A statistical software program (Stata, version 12; StataCorp LP) was used to perform these analyses. Group-based trajectory modeling is designed to identify groups of individuals who are following similar trajectories of a single outcome variable.^33^ Multitrajectory modeling extends the basic model by defining trajectory groups in terms of multiple outcome variables.^29^ Both the basic model and the multitrajectory generalization are applications of finite mixture modeling. The multitrajectory method applied herein combined the trajectories of physical aggression based on mother ratings (ages 1.5-8 years), the trajectories of physical aggression based on teacher ratings (ages 6-13 years), and the trajectories of physical aggression based on self-ratings (ages 10-13 years). Therefore, the multitrajectories are the product of combined estimation procedures for each group’s trajectories across the 3 raters. Two criteria (the Bayesian information criterion [BIC] score and substantive significance) were used to determine the number of groups to include in the multitrajectory modeling.^33^ Multinomial logistic regression models were used to estimate the association between risk factors—such as parents’ education, household income, mother’s age at first childbirth, family structure, parents’ depression, number of siblings, and parents’ history of antisocial behavior—and the chronic trajectory of physical aggression.

Like most maximum likelihood models, multitrajectory modeling assumes that data are missing at random. Under this assumption, there is no constraint on the number of missing observations that individuals may have to be included in the estimation sample. Therefore, we included a condition requiring individuals to have at least 10 observations across the 16 observations to be included in the estimation sample. In addition, we checked using single-trajectory modeling whether the attrition at later periods of data collection was systematically different for a given type of physical aggression trajectory or was equally distributed. We estimated the posterior probability or group membership for each period for mother ratings and performed a cross-tabulation between group membership at each period, with nonresponse or participating category at the last 2 periods (ages 12 and 13 years). The results showed that the attrition took place equally in all trajectory groups. Finally, we also estimated the model without having the restriction of at least 10 of 16 observations per person to see if the results changed by the removal of this restriction.

## Results

Among 2223 participants, 51.2% were boys and 91.2% were of white race/ethnicity. The development of children’s physical aggression was examined with 3 raters across 16 observations at different time points from age 18 months to age 13 years. Table 1 lists the means (SDs) of physical aggression at all assessment times for the whole sample and by sex. It also summarizes the results of *t* tests for sex differences. As expected, significant sex differences were observed across all assessment times, with girls having lower levels of physical aggression and lower variance, especially according to teacher ratings. The highest mean level of physical aggression was at age 3.5 years for both boys and girls. Therefore, according to mother ratings, teacher ratings, and self-ratings, the frequency of physical aggressions increased from age 1.5 years (2039 [91.7%]) to age 3.5 years (1941 [87.3%]) and then substantially decreased until age 13 years (1228 [55.2%]).

**Table 1.** Attrition and Sex Differences on 16 Observations Among 2223 Participants

Age, y	Whole Sample		Boys		Girls		Sex Difference (95% CI)
	No. (%)	Mean (SD) of PA^a^	No.	Mean (SD) of PA^a^	No.	Mean (SD) of PA^a^	
Mother ratings							
1.5	2039 (91.7)	0.69 (0.93)	1030	0.74 (0.98)	1009	0.63 (0.87)	−2.64 (−0.19 to −0.03)^b^
2.5	1993 (89.7)	0.99 (1.06)	1002	1.10 (1.09)	991	0.88 (1.00)	−4.88 (−0.32 to −0.14)^c^
3.5	1941 (87.3)	1.37 (1.36)	976	1.54 (1.42)	965	1.20 (1.27)	−5.57 (−0.46 to −0.22)^c^
4.5	1937 (87.1)	1.09 (1.25)	972	1.27 (1.33)	965	0.92 (1.14)	−6.31 (−0.46 to −0.25)^c^
5	1757 (79.0)	1.06 (1.26)	873	1.24 (1.34)	884	0.88 (1.15)	−5.87 (−0.47 to −0.23)^c^
6	1488 (66.9)	0.98 (1.23)	731	1.21 (1.30)	757	0.75 (1.11)	−7.41 (−0.59 to −0.34)^c^
8	1448 (65.1)	0.92 (1.25)	695	1.15 (1.36)	753	0.71 (1.10)	−6.74 (−0.56 to −0.31)^c^
Teacher ratings							
6	944 (42.5)	0.57 (1.23)	440	0.97 (1.48)	504	0.21 (0.79)	−10.03 (−0.91 to −0.61)^c^
7	1264 (56.9)	0.66 (1.29)	596	1.12 (1.55)	668	0.25 (0.81)	−12.72 (−1.00 to −0.74)^c^
8	1206 (54.3)	0.54 (1.17)	561	0.90 (1.45)	645	0.21 (0.70)	−10.83 (−0.82 to −0.57)^c^
10	940 (42.3)	0.49 (1.10)	435	0.89 (1.42)	505	0.14 (0.52)	−11.09 (−0.88 to −0.62)^c^
12	948 (42.6)	0.50 (0.90)	449	0.65 (1.17)	499	0.08 (0.37)	−10.22 (−0.67 to −0.45)^c^
13	786 (35.4)	0.18 (0.71)	321	0.38 (0.99)	465	0.04 (0.34)	−6.75 (−0.43 to −0.24)^c^
Self-ratings							
10	1299 (58.4)	0.66 (1.08)	613	0.95 (1.26)	686	0.40 (0.80)	−9.62 (−0.67 to −0.44)^c^
12	1346 (60.5)	0.69 (0.93)	643	0.82 (1.02)	703	0.56 (0.81)	−5.18 (−0.36 to −0.16)^c^
13	1228 (55.2)	0.63 (0.89)	564	0.75 (0.98)	664	0.53 (0.78)	−4.33 (−0.32 to −0.12)^c^

Abbreviation: PA, physical aggression.

^a^ The minimum score for PA was 0, and the maximum score was 6, with higher scores indicating greater PA.

^b^ Statistically significant at *P* < .01.

^c^ Statistically significant at *P* < .001.

### Multitrajectory Analyses for Boys

**Figure 1.**
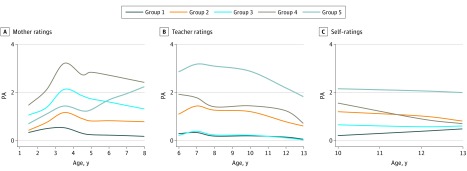
Physical Aggression (PA) Trajectories Among 730 Boys Likert-type scale score range of 0 to 6 is transformed to 0 to 4 to highlight the differences between trajectories.

The BIC score for boys was maximized for a 4-group model. However, this 4-group model did not produce a distinct chronic physical aggression trajectory, while the 5-group model led to a clearer chronic trajectory (Figure 1). The BIC score differences between the 4-group model and the 5-group model were not notable enough to make a strong case for a 4-group model (eTable in the Supplement). Table 2 compares the adequacy of the different models. In addition, the 5-group model for physical aggression was consistent with a previous multitrajectory study^29^ among the same population, which oversampled boys from low socioeconomic environments. Given the focus of the present study on identifying the small group of boys who follow a chronic physical aggression trajectory, the substantive advantages of the 5-group model outweigh the small drop in explanatory power from the 4-group model as measured by the BIC score (a further discussion of model selection is given by Nagin^33^). The 5-group model performs well on the model adequacy tests laid out by Nagin.^33^ Specifically, the average posterior probability of group membership ranges between 79.5% and 89.4%, which exceeds the 0.7 threshold for all group assignments based on the maximum posterior probability assignment rule; the odds of correct classification compared with the odds of correct classification based on random assignment exceed 5 for each trajectory group; and the estimated probability of the trajectory group is always close to the proportion assigned to the group (Table 2).

**Table 2.** Quality of the Classification Operated by the Models to Compare Adequacy^a^

Trajectory	Boys (n = 730)					Girls (n = 781)				
	No.	APP	OCC	Estimated %	Assigned %	No.	APP	OCC	Estimated %	Assigned %
**3-Group Model**										
1	323	93.4	18.0	44.2	43.7	413	85.8	5.4	52.9	50.1
2	247	85.9	11.9	33.8	32.9	169	70.7	8.8	21.6	22.3
3	160	85.9	21.8	21.9	23.4	199	93.5	42.1	25.5	27.6
**4-Group Model**										
1	257	87.9	13.3	35.2	32.9	201	82.9	14.0	25.7	23.1
2	153	82.6	17.9	21.0	22.0	274	83.3	9.3	35.1	37.3
3	187	84.7	16.1	25.6	27.1	250	83.9	11.1	32.0	32.1
4	133	86.9	29.8	18.2	18.0	56	85.6	77.2	7.2	7.6
**5-Group Model**										
1	190	89.4	23.9	26.0	26.4	193	84.6	16.7	24.7	22.3
2	223	79.5	8.8	30.5	29.0	110	78.3	22.1	14.1	16.6
3	150	78.5	14.1	20.5	20.3	195	76.7	9.9	25.0	23.5
4	123	85.5	29.0	16.8	17.5	199	82.8	14.1	25.5	26.5
5	44	83.5	78.7	6.0	6.8	84	86.5	52.9	10.8	11.1

Abbreviations: APP, average posterior probability; OCC, odds of correct classification.

^a^ After implementing the restriction function to delete the participants with less than 10 of 16 observations, the number of participants is 1511.

Group 1 included 26.0% (190 of 730) of boys characterized by very low levels of physical aggression according to ratings by mothers, teachers, and self-reports from ages 1.5 to 13 years. Group 2 included 30.5% (n = 223) of boys who displayed low to medium stable levels of physical aggression according to ratings by mothers, teachers, and self-reports. Group 3 included 20.5% (n = 150) of boys; they were rated by mothers as having medium levels of physical aggression from ages 1.5 to 8 years, by teachers as having low levels of physical aggression from ages 6 to 13 years and by self-reports as having medium levels of physical aggression between ages 10 and 13 years. Group 4 included 16.8% (n = 123) of boys; they were rated by mothers as having the highest level of physical aggression from ages 1.5 to 8 years and by teachers and self-reports as having medium levels of physical aggression from ages 6 to 13 years. Group 5 included 6.0% (n = 44) of boys; they were rated by their mothers as having medium to increasing levels of physical aggression from ages 1.5 to 8 years, while teacher ratings and self-reports from ages 6 to 13 years placed them on the highest levels of physical aggression.

### Multitrajectory Analyses for Girls

**Figure 2.**
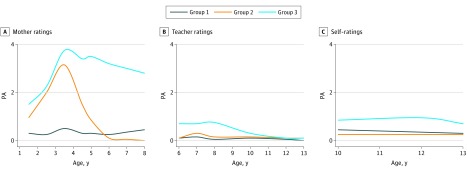
Physical Aggression (PA) Trajectories Among 781 Girls Logit score range of 0 to 1 is shown from 0 to 0.8 to highlight the distinction between trajectories.

Because of the small variance in physical aggression scores for girls, especially according to teacher ratings and self-ratings, we were unable to estimate meaningful models based on the Likert-type scale data. Therefore, we transformed the responses from a Likert-type scale to a binary format by recoding scores from 0 to 1.5 as 0 (not aggressive) and from 1.6 to 6 as 1 (aggressive). Using this recoding, we were successful in estimating a 3-group model, and the BIC score for the 3-group model was maximized as well (Figure 2 and eTable in the Supplement). Group 1 included 52.9% (413 of 781) of girls. Mother ratings, teacher ratings, and self-ratings all converged to indicate that they were on a low physical aggression trajectory. Group 2 included 21.6% (n = 169) of girls. Ratings by mothers, teachers, and self-reports indicated that these girls had low levels of physical aggression from ages 5 to 13 years, after having been rated by mothers as being at high levels of physical aggression between ages 1.5 and 4 years. Group 3 included 25.5% (n = 199) of girls who were rated as having the highest frequency of physical aggressions by mothers from ages 1.5 to 8 years, by teachers from ages 6 to 9 years, and by self-reports from ages 10 to 13 years. We also examined the model adequacy test for 3-group trajectory of girls’ physical aggression, and all indexes—including the average posterior probability of group membership, the odds of correct classification compared with the odds of correct classification based on random assignment, and the proximity of the estimated probability of trajectory group and the proportion assigned to the group—exceeded the acceptable threshold (Table 2).

### Dropout Consequences

As mentioned earlier, in the trajectory modeling, we listwise deleted individuals with less than 10 of 16 observations for the purpose of limiting the consequences of missing values on trajectories. We calculated the trajectories without the use of this constraint to examine whether the size and shape of the trajectories changed significantly. The model change was negligible for girls’ trajectories: neither the distribution of group membership nor their developmental course was materially altered. As with girls, the developmental trajectories of boys based on the full sample were almost identical to those based on the use of the constraint function. However, there was a material reduction in the size of the chronic trajectory from 6.0% to 3.1%, accompanied by an increase in group 3 from 20.5% to 21.9% and in group 4 from 16.8% to 18.7%. We concluded from this sensitivity analysis that our main findings were not altered by our modeling decision to limit the analysis to individuals with at least 10 of 16 observations.

### Differences in Family Characteristics Between Those in the High Physical Aggression Trajectories vs the Others

As summarized in Table 3, the boys rated as highest on physical aggression by teachers and self-reports from ages 6 to 13 years (group 5 [6.0%]) were significantly different from the boys in groups 1 to 3 for father’s education, household income, socioeconomic status, and mother’s antisocial behavior during her own adolescence. The same comparison of group 4 (16.8%) with groups 1 to 3 indicated similar significant differences, plus significant differences for mother’s education, mother’s young age at first childbirth, mother’s depression, and the number of siblings at the target child’s birth. In general, boys on the chronic trajectories had parents with lower education and higher depression, mothers with a young age at first childbirth, and lower socioeconomic status.

**Table 3.** Differences in Family Characteristics on 12 Family Risk Factors Between Those in the High Physical Aggression Trajectories vs the Others Based on Logistic Regressions

Variable^a^	Boys (n = 730)				Girls (n = 781)			
	Group 4 vs Groups 1, 2, and 3		Group 5 vs Groups 1, 2, and 3		Group 2 vs Group 1		Group 3 vs Group 1	
	β (SE)	*z* Score	β (SE)	*z* Score	β (SE)	*z* Score	β (SE)	*z* Score
Mother’s education	−0.69 (0.21)	−3.27^b^	−0.61 (0.33)	−1.83	0.03 (0.20)	−0.14	−0.78 (0.19)	−4.13^c^
Father’s education	−0.81 (0.21)	−3.71^c^	−1.10 (0.34)	−3.20^b^	0.14 (0.21)	0.69	−0.69 (0.19)	−3.50^c^
Household income	−0.89 (0.21)	−4.10^c^	−0.69 (0.34)	−2.00^d^	0.15 (0.20)	0.74	−0.90 (0.19)	−4.76^c^
Mother’s age at first childbirth	−2.59 (0.93)	−2.78^b^	−2.62 (1.47)	−1.79	1.32 (0.74)	−1.77	−0.52 (0.70)	−0.74
Family structure (intact)	0.06 (0.02)	2.46^d^	0.03 (0.04)	0.68	0.00 (0.02)	0.20	0.03 (0.02)	1.54
Mother’s depression	0.48 (0.12)	3.74^c^	0.27 (0.20)	1.29	0.25 (0.11)	2.13^d^	0.40 (0.11)	3.64^c^
Father’s depression	0.13 (0.10)	1.44	0.13 (0.15)	0.91	0.07 (0.09)	0.74	0.19 (0.09)	2.12^d^
Socioeconomic status	−0.39 (0.09)	−4.30^c^	−0.41 (0.14)	−2.85^b^	−0.09 (0.09)	−1.04	−0.43 (0.08)	−5.11^c^
No. of siblings at 5 mo	0.17 (0.08)	1.98^d^	0.03 (0.13)	0.24	0.25 (0.08)	−2.98^b^	0.42 (0.08)	5.40^c^
No. of siblings at 17 mo	0.26 (0.08)	3.21^b^	0.10 (0.13)	0.75	0.27 (0.08)	3.36^b^	0.46 (0.07)	6.17^c^
Mother’s antisocial behavior during her own adolescence	0.37 (0.10)	3.89^c^	0.29 (0.15)	1.96^d^	−0.10 (0.08)	−1.21	0.13 (0.07)	1.72
Father’s antisocial behavior during his own adolescence	0.02 (0.10)	0.26	0.29 (0.16)	1.83	0.08 (0.09)	0.86	0.35 (0.09)	4.00^c^

^a^ All variables measured at 5 mo unless otherwise indicated.

^b^ Statistically significant at *P* < .01.

^c^ Statistically significant at *P* < .001.

^d^ Statistically significant at *P* < .05.

For girls, group 3 (rated the most physically aggressive from ages 1.5 to 13 years according to all raters) was significantly different from group 1 on all measured family characteristics except for mother’s age at first childbirth, family structure, and mother’s antisocial behavior during her own adolescence (Table 3). Group 2 girls—who were rated by mothers as having high levels of physical aggression from ages 1.5 to 4 years but were rated with low levels of physical aggression afterward according to all raters—were significantly different from group 1 on mother’s depression and the number of siblings at age 5 months and age 17 months.

## Discussion

The objective of this study was to identify (for the first time, to our knowledge) the developmental trajectories of physical aggression for boys and girls from infancy to early adolescence simultaneously using reports from mothers (ages 17 months to 8 years), teachers (ages 6-13 years), and the children themselves (ages 10-13 years). We also examined early risk factors for high physical aggression trajectories.

As expected, the overall frequency of physical aggressions increased from ages 1.5 to 3.5 years and then substantially decreased. In general, there was discontinuity in the frequency of physical aggressions by girls, highlighted by an important reduction from early childhood to middle childhood, while there was relative continuity for boys, with decreasing scores over time except for group 5, which showed a substantial increase from ages 5 to 7 years. The differences in the frequency of physical aggressions reported by mothers and teachers between ages 6 and 8 years were especially important for girls on the highest aggression trajectory. This outcome may reflect a real difference in physically aggressive behavior at home with siblings and at school with peers.

Although there are large sex differences in the use of physical aggression throughout development,^25,34^ most published studies on the developmental trajectories of physical aggression with large samples pooled boys and girls together.^4,6,10,11,16,21,27,35^ Based on the limited number of longitudinal studies that traced the developmental trajectories of physical aggressions separately for boys and girls with population samples, we expected to observe important sex differences.^3,7,23^ The present study covers a longer time span and simultaneously uses 3 different sources of information. The results showed substantial sex differences from 3 different perspectives, including the number of trajectories, the form of the trajectories, and the number of individuals in the different trajectories.

The multitrajectory analysis for girls identified the expected, consistent high physical aggression trajectory (group 3) from mother ratings (between ages 1.5 and 8 years) and from teacher ratings and self-ratings (ages 6-13 years). As expected also, their mean level of physical aggression at each assessment was substantially lower than that of the boys on a high physical aggression trajectory. However, the girls on the highest physical aggression trajectory represented almost one-third of the female sample (25.5%). It is clear that we are not dealing with a small part of the population as we are with group 5 in the sample of boys. Nonetheless, when we compared the girls on the high (group 3) and low (group 1) trajectories for the 12 family risk factors measured 5 months after the child’s birth, the girls on the high trajectory were significantly different from those on the low trajectory for 9 of these 12 risk factors: mother’s and father’s education, household income, maternal and paternal depression, socioeconomic status, number of siblings at birth and at 17 months, and father’s antisocial behavior during his own adolescence. These results indicate that risk factors during pregnancy and early childhood are good predictors of a substantial number of girls who will have more problems with the use of physical aggression from early childhood to adolescence. As shown in a previous study^15^ with the same sample, these girls are at high risk for important problems with school achievement, nicotine use, early pregnancy, and intimate partner violence. The multitrajectory results for the boys also led to a generally unexpected result in our study. The 6.0% of boys who were on the high physical aggression trajectory according to teacher ratings and self-ratings between ages 6 and 13 years (group 5) were the only group that did not follow the expected trajectory during early childhood: they were not on the highest physical aggression trajectory from ages 1.5 to 8 years according to mother ratings. In fact, they were on a middle-level trajectory up to age 5 years, although mother ratings indicated a steady increase in the frequency of physical aggressions from ages 1.5 to 8 years. If we can rely on the maternal reports, these results for group 5 indicate a substantial increase in the frequency of physical aggressions starting at school entry and peaking at age 7 years. The family risk factors that were assessed at 5 months after the child’s birth indicate that these boys were (from the start) living in family conditions of very high risk. They were significantly different from the 3 lowest trajectory groups on 5 of the 12 family risk factors. The group of boys that were on the highest physical aggression trajectory from ages 1.5 to 3 years according to mothers (group 4) went on a decreasing trajectory: by age 13 years, their frequency of physical aggressions was similar to that of the low groups (1, 2, and 3). Based on the comparison of family characteristics assessed at 5 months after the child’s birth (Table 3), group 4 boys were significantly different from groups 1, 2, and 3 on 10 of the 12 family risk factors.

The present study indicates that boys and girls have different patterns of the development of physical aggression. One of the important sex differences is that girls on a high physical aggression trajectory during early childhood (according to mothers) remain on the high physical aggression trajectory from ages 6 to 13 years according to teachers and self-reports. However, boys on the high trajectory according to teachers and self-reports were not rated on the high trajectory by mothers during early childhood.

One explanation for this difference in the results between boys and girls is that mothers of boys living in high-risk families may not be the most reliable raters of their son’s physical aggression frequency. This hypothesis could be verified by comparing physical aggression ratings by mothers and by child care workers when the boys are in child care. However, an important challenge is that children from at-risk families are less likely to be attending child care services.^36^ Studies based on information gathered by health care professionals during well-child care visits could also increase our understanding of the source of what appears to be a difficulty by some mothers to identify their son’s physical aggression problems before entering elementary school. A further question concerning the same issue for future research is why mothers during early childhood mothers appear to be good at identifying the girls but not the boys who will have high levels of physical aggression according to teachers and self-ratings from ages 6 to 13 years. One hypothesis is that mothers in at-risk families more easily understand the inappropriate aggressive behavior of their daughters than they do of their sons.^37^

### Limitations

Despite the strength and importance of our findings, our study has some limitations. First, the sample was primarily children of white race/ethnicity living in the Canadian province of Quebec. Similar analyses should be done with comparable data sets from other cultural environments. Second, the attrition was high and was related in part to low socioeconomic status. Although the attrition analyses provided some evidence that the findings were not altered by the attrition, more comprehensive analysis of the association of socioeconomic background with trajectories of physical aggression will probably shed more light on the differences in trajectories of physical aggression across different social strata. Third, this study is limited to the responses of a parent (mostly mothers) to the child’s physical aggression. Assessments from fathers and child care staff should help increase the reliability of preschool assessments of the early development of physical aggression that is needed for targeting preventive interventions.

Trajectory modeling purports to approximate an underlying, unknown continuous distribution of developmental patterns for the purpose of studying their salient features. That is, the trajectories are not fixed and should only be treated as explanatory models for possible developmental outcomes. Similarly, the results of the present study should be treated as explanatory models that suggest important directions for future research.

## Conclusions

There is a need for studies designed to investigate why some boys on a high physical aggression trajectory according to teachers and self-reports are not perceived by their mothers as being physically aggressive during the preschool years. Assessments by health care professionals during well-child care visits and by child care educators could be used to compare with maternal reports. Also needed are studies with similar data sets to replicate our findings with multitrajectory analyses. Experiments using preventive interventions with at-risk families during pregnancy and early childhood will enable us to find effective ways of preventing high physical aggression trajectories for boys and girls living in high-risk families.

## Notes

Supplement. **eTable.** Comparison of the Fit Statistics of Different Multitrajectory Models
