# Supplementary material for: Risk Factors Associated With Boys’ and Girls’ Developmental Trajectories of Physical Aggression From Early Childhood Through Early Adolescence
Source: JAMA Netw Open. 2018 Dec 28;1(8):e186364. doi: 10.1001/jamanetworkopen.2018.6364 (PMC6324348; doi:10.1001/jamanetworkopen.2018.6364)
Supplement: Supplement. — eTable. Comparison of the Fit Statistics of Different Multitrajectory Models [file jamanetwopen-1-e186364-s001.pdf]

Supplementary Online Content

Teymoori A, Côté SM, Jones BL, et al. Assessment of risk factors associated with the development of physical aggression in boys and girls from childhood through early adolescence. *JAMA Netw Open*. 2018;1(8):e186364. doi:10.1001/jamanetworkopen.2018.6364

eTable. Comparison of the Fit Statistics of Different Multitrajectory Models

This supplementary material has been provided by the authors to give readers additional information about their work.

© 2018 Teymoori A et al. *JAMA Network Open*.

eTable. Comparison of the Fit Statistics of Different Multitrajectory Models

		Boys						Girls				
		BIC	Bayes factor (a)	2*BIC diff (b)	AIC	% smaller class		BIC	Bayes factor (a)	2*BIC diff (b)	AIC	% smallest class
2-class solution		-23441.07			-23390.81	26.5		-8997.31			-8968.21	24.3
3-class solution		-23219.46	0.0000	443.22	-23145.38	23.4		-8822.14	0.0000	350.34	-8777.16	27.6
4-class solution		-23006.42	0.0000	426.08	-22916.48	18		-17231.27	0.0000	-16818.26	-17162.49	7.6
5-class solution		-23014.93	4964.16	-17.02	-22909.11	6.8		-17172.35	0.0000	117.84	-17095.63	11.1
6-class solution		No convergence						-17094.91	0.0000	154.88	-16999.67	7.1

a. exp([BIC model N-1 classes] - [BIC model N classes])

b. 2 x ([BIC model N classes] - [BIC model N -1 classes])

© 2018 Teymoori A et al. *JAMA Network Open*.
